# *Streptococcus pneumoniae* pneumolysin and neuraminidase A convert high-density lipoproteins into pro-atherogenic particles

**DOI:** 10.1016/j.isci.2021.102535

**Published:** 2021-05-12

**Authors:** Shahan Syed, Eija Nissilä, Hanna Ruhanen, Satoshi Fudo, Meztlli O. Gaytán, Sanna P. Sihvo, Martina B. Lorey, Jari Metso, Katariina Öörni, Samantha J. King, Oommen P. Oommen, Matti Jauhiainen, Seppo Meri, Reijo Käkelä, Karita Haapasalo

**Affiliations:** 1 Department of Bacteriology and Immunology, University of Helsinki, 00014 Helsinki, Finland; 2 Molecular and Integrative Biosciences Research Programme, Faculty of Biological and Environmental Sciences, University of Helsinki, 00014 Helsinki, Finland; 3 Helsinki University Lipidomics Unit (HiLIPID), Helsinki Institute for Life Science (HiLIFE) and Biocenter Finland, Helsinki 00014, Finland; 4 Center for Microbial Pathogenesis, Abigail Wexner Research Institute at Nationwide Children's Hospital, Columbus, OH 43205, USA; 5 Wihuri Research Institute, 00290 Helsinki, Finland; 6 Minerva Foundation Institute for Medical Research, Biomedicum, 00290 Helsinki, Finland; 7 Department of Pediatrics, The Ohio State University, Columbus, OH 43210, USA; 8 Bioengineering and Nanomedicine Lab, Faculty of Medicine and Health Technology and BioMediTech Institute, Tampere University, 33720 Tampere, Finland; 9 Genomics and Biomarkers Unit, National Institute for Health and Welfare, Helsinki, Finland

**Keywords:** Cardiovascular medicine, Microbiology

## Abstract

High-density lipoproteins (HDLs) are a group of different subpopulations of sialylated particles that have an essential role in the reverse cholesterol transport (RCT) pathway. Importantly, changes in the protein and lipid composition of HDLs may lead to the formation of particles with reduced atheroprotective properties. Here, we show that *Streptococcus pneumoniae* pneumolysin (PLY) and neuraminidase A (NanA) impair HDL function by causing chemical and structural modifications of HDLs. The proteomic, lipidomic, cellular, and biochemical analysis revealed that PLY and NanA induce significant changes in sialic acid, protein, and lipid compositions of HDL. The modified HDL particles have reduced cholesterol acceptor potential from activated macrophages, elevated levels of malondialdehyde adducts, and show significantly increased complement activating capacity. These results suggest that accumulation of these modified HDL particles in the arterial intima may present a trigger for complement activation, inflammatory response, and thereby promote atherogenic disease progression.

## Highlights

• *S. pneumoniae* molecules PLY and NanA target human high-density lipoprotein (HDL).
• These interactions result in major modifications in the HDL proteome and lipidome.
• Microbially modified HDL activates humoral and cell-mediated innate immune responses.
• The activated immune response mediates formation of pro-atherogenic epitopes on HDL.

## Graphical abstract

**Figure:**
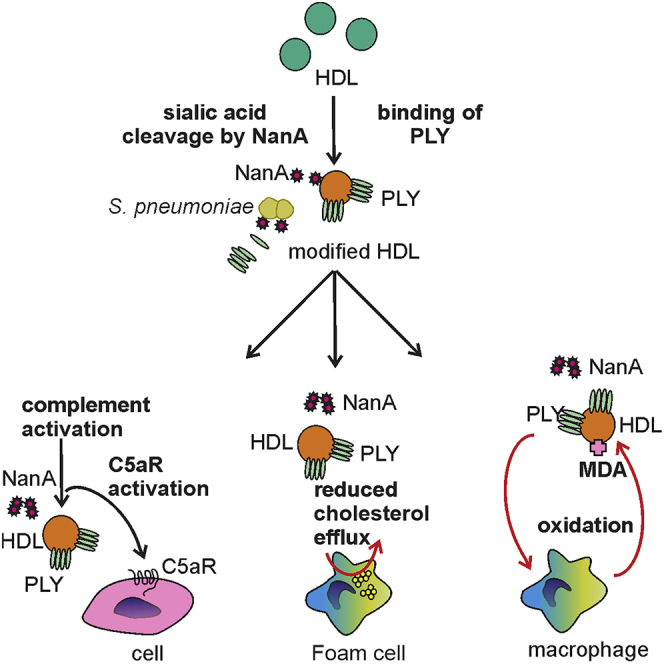

## Introduction

*Streptococcus pneumoniae* is an important pathogen that typically causes otitis media, sinusitis, pneumonia, and more rarely severe infections such as sepsis and meningitis. Despite the availability of vaccines, pneumococcus is still one of the most common causes of hospitalization in adults, especially the elderly (Gil-Prieto et al., 2016). *S. pneumoniae* possesses virulence factors such as pneumolysin (PLY) and neuraminidase A (NanA). PLY is a cholesterol-dependent cytolysin that lyses human cells, while NanA removes terminal sialic acids including those on host cell surfaces and sensitizes cells for attack by PLY (Burnaugh et al., 2008; Kanclerski and Mollby, 1987; Syed et al., 2019).

The role of cellular cholesterol efflux mediated by high-density lipoprotein (HDL) is crucial to prevent formation of foam cells, generation of pro-inflammatory macrophages, and inflammation, all of which play a crucial role in the pathogenesis of atherosclerosis (Paoletti et al., 2004). HDL also possesses anti-inflammatory activities and binds and neutralizes microbial molecules such as lipopolysaccharides (LPSs) and lipoteichoic acids (Blauw et al., 2020; Gordon et al., 2011; Khovidhunkit et al., 2004; Pirillo et al., 2015a). Importantly, inflammation caused by acute microbial infection or coronary artery disease launches significant changes in lipid metabolism and in the protein and lipid composition of HDL. These chemical, compositional, and structural changes can transform atheroprotective HDL into dysfunctional pro-atherogenic and pro-inflammatory particles with attenuated cholesterol acceptor and anti-inflammatory activities (Pirillo et al., 2015b; Smith, 2010). This suggests a role for microbial molecules in modifying the anti-inflammatory effects of HDL. The two main subfractions of HDL, HDL-2 and HDL-3, differ in their density, size, protein, and lipid composition (Kaji, 2013). Of these, HDL-2 is more susceptible to oxidation, and the resulting modifications may impair its function in mediating cellular cholesterol efflux (Paavola et al., 2017).

We show here that *S. pneumoniae* PLY and NanA interact with HDL. Binding of PLY to HDL leads to reduced PLY and NanA-mediated hemolytic activity and simultaneously to formation of modified HDL particles that display changed protein, lipid, and sialic acid composition which are associated with cardiovascular diseases (Cardner et al., 2020; Okada et al., 2019). These modified HDL particles are more susceptible to complement attack and possess reduced capacity to mediate cholesterol efflux. These results provide a novel explanation of how microbial infections could be linked to atherosclerosis by modifying HDL and potentially reducing its atheroprotective properties.

## Results

### HDL inhibits PLY cytolytic activity

**Figure 1:**
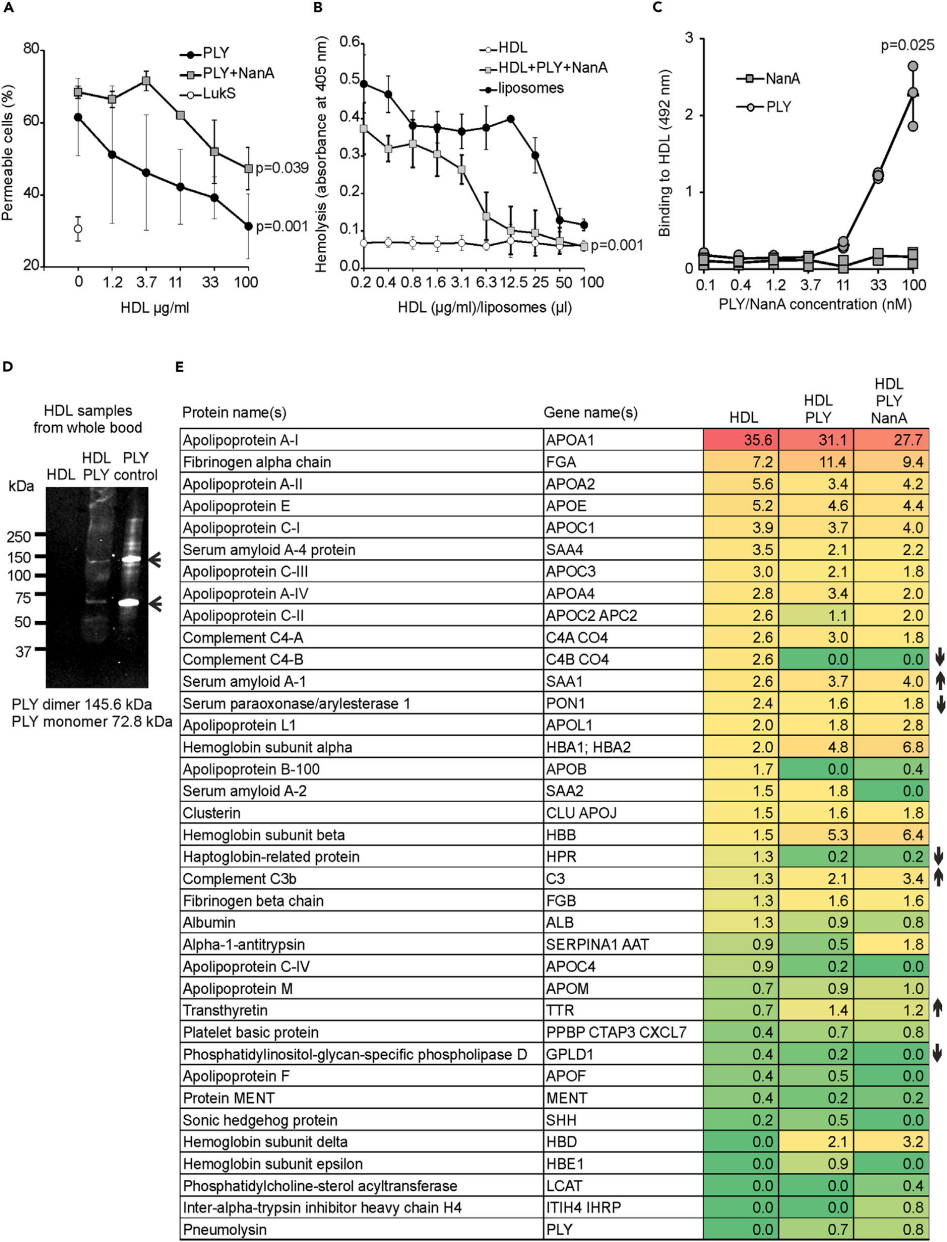
Interaction between HDL and *S. pneumoniae* molecules PLY and NanA reduces PLY-mediated cytolysis and leads to HDL modification *ex vivo* (A) THP-1 cells incubated with increasing concentrations of HDL-2 with or without 100 nM PLY or 100 nM PLY and 800 nM NanA. LukS was used as a negative control for cell lysis. Permeability is shown as the percentage of cells staining positive for 4′,6-diamidino-2-phenylindole in flow cytometry. See also Figures S1A and S1B. (B) Human red blood cells incubated with 10 nM PLY and 800 nM NanA. The effect of HDL-2 on the lytic activity of PLY was tested by the addition of increasing concentrations of HDL-2. Cholesterol-mediated inhibition was analyzed with increasing concentrations of liposomes. A sample with only HDL-2 was used as a negative control. (C) Enzyme-linked immunosorbent assay indicating binding of PLY to HDL-2. See also Figures S1C and S1D. (D) Western blot of total HDL samples, isolated from plasma, incubated with (HDL + PLY) or without PLY (HDL). PLY control shows the size of the PLY monomer and dimer in sodium dodecyl sulfate-polyacrylamide gel electrophoresis (SDS-PAGE). (E) Total HDL samples isolated from *ex vivo* experiment analyzed by MS/MS. See also Table S1. Numbers are shown as the percentage of peptide spectrum matches (PSMs) of all identified proteins with a score of >10 or PSMs >4 in the HDL sample. Increase or reduction in the protein content in plasma-isolated HDL samples from the blood incubated with PLY and NanA compared to the sample without microbial molecules is marked with arrows. The detected statistically significant differences (p < 0.05) between highest and lowest ([A] and [B]) HDL or (C) PLY concentrations were calculated using one-way analysis of variance (ANOVA) followed by Dunnett's non-parametric multiple comparison test. Error bars indicate standard deviation (SD) values calculated from three repeated experiments.

Because HDL is known to bind to several microbial molecules (Figueiredo et al., 2003; Levine et al., 1993; Surewaard et al., 2012), we analyzed whether HDL could inhibit PLY-mediated hemolysis. While NanA increased PLY-mediated lysis especially on white blood cells (Figures S1A and S1B), the cytolytic effect of PLY and NanA on both human red and white blood cells was significantly inhibited by the addition of HDL-2 (Figures 1A and 1B). Similarly, the presence of cholesterol-containing liposomes caused dose-dependent inhibition of PLY-mediated hemolysis. These results suggest that the inhibitory effect of HDL-2 on PLY hemolytic activity is due to the interaction of PLY with HDL surface unesterified cholesterol, which would reduce binding of the toxin to cell surfaces, thereby reducing PLY-mediated hemolysis.

### HDL interaction with PLY and NanA leads to HDL modification *ex vivo*

To understand the mechanism behind the HDL-2-mediated inhibition of PLY, we first analyzed whether PLY binds to HDL. As we expected, PLY but not NanA bound to HDL-2 in a dose-dependent fashion (Figure 1C). Interestingly, PLY bound less to rabbit HDL, indicating species specificity of this toxin as observed for other cytolysins (Figure S1C) (Nagamune et al., 1997; Spaan et al., 2017). Binding of PLY to HDL also showed subclass specificity since it bound significantly less to HDL-3 than to HDL-2 (Figure S1D). Also, *ex vivo* binding of both the PLY monomer and dimer was detected on the isolated total HDL fraction (Figure 1D) indicating PLY oligomerization (Gilbert et al., 1998). The presence of PLY and absence of NanA on the isolated HDL particles from the *ex vivo* assay was verified by mass spectrometry (MS). An absence of C4B in the PLY-treated samples further verified specific binding of PLY to the 3β-hydroxyl group of cholesterol which is the ligand for both complement C4B and cholesterol-dependent cytolysins (Law et al., 1984) (Figure 1E and Table S1). The proteomic profiles also revealed differences between the HDL particles isolated from plasma that were incubated with or without PLY and NanA. Increases in hemoglobin subunits and complement C3 reflected lytic function of PLY and susceptibility of the HDL to complement mediated attack, respectively. In contrast, the reduction in serum paraoxonase (PON-1) and increase in serum amyloid A1 indicated the loss of functionality and cardioprotective effects of HDL (Shao and Heinecke, 2018).

**Figure 2:**
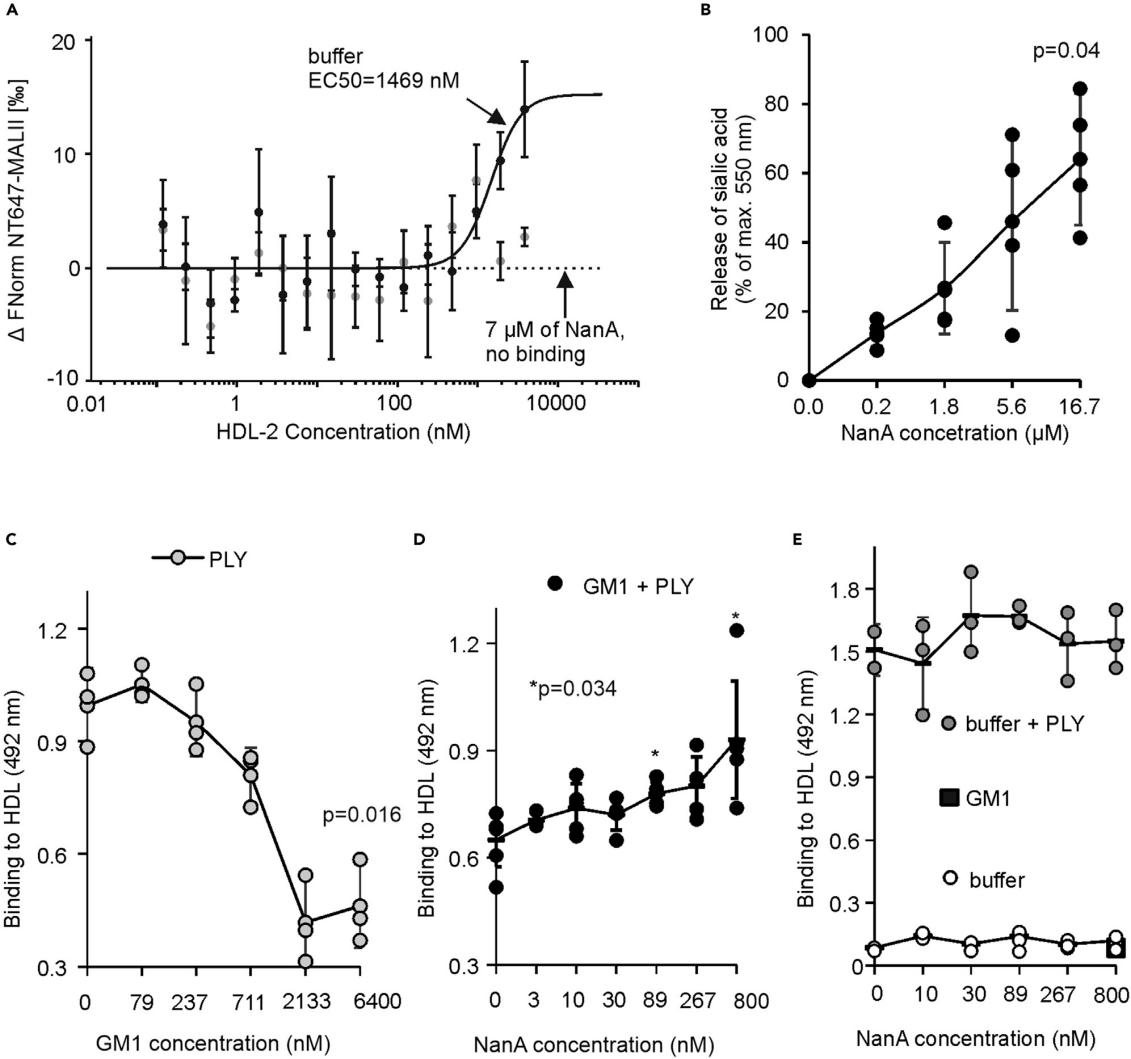
NanA activity exposes HDL-2 for PLY binding (A) Absence of sialic acids on HDL-2 incubated with NanA shown by loss of binding of MALII in microscale thermophoresis (affinity 1469 nM in the presence of sialic acids). (B) Release of free sialic acid in the presence of NanA. Release of sialic acids is calculated as the percentage of maximum absorbance measured without NanA. (C) Increasing concentrations of GM1 inhibit PLY binding to HDL-2. (D) NanA treatment of HDL-2 with 6 μM GM1 causes a concentration-dependent increase in PLY binding (GM1+PLY). (E) HDL-2 without GM1 (buffer + PLY) shows no increase in PLY binding. HDL-2 with (GM1) or without GM1 and PLY (buffer) shows no binding of NanA. The detected statistical significance (p < 0.05) the between highest and lowest (B, D) NanA and (C) GM1 and concentrations was calculated using one-way ANOVA followed by Dunnett's non-parametric multiple comparison test. Error bars showing SDs are calculated from at least three biological replicates.

Incubation of HDL with NanA resulted in the release of terminal sialic acids from HDL as detected by loss of binding of the sialic acid-binding lectin MALII and by the detection of free sialic acids (Figures 2A and 2B). These results suggest that HDL particles are susceptible to the enzymatic activity of NanA. To further understand the concerted function of PLY and NanA in HDL modification, we coated HDL with monosialotetrahexosylganglioside (GM1). The presence of GM1 on HDL-2 significantly reduced binding of PLY to HDL-2 as compared to binding to non-GM1-coated HDL-2 (Figure 2C). While no binding of NanA to HDL-2 could be detected, regardless of the presence of GM1, the addition of NanA increased binding of PLY to GM1-coated HDL-2 (Figures 2D and 2E). These results indicate that the cleavage of terminal sialic acids by NanA exposes unesterified cholesterol on the surface of HDL for PLY binding.

### PLY and NanA modify HDL particles

**Figure 3:**
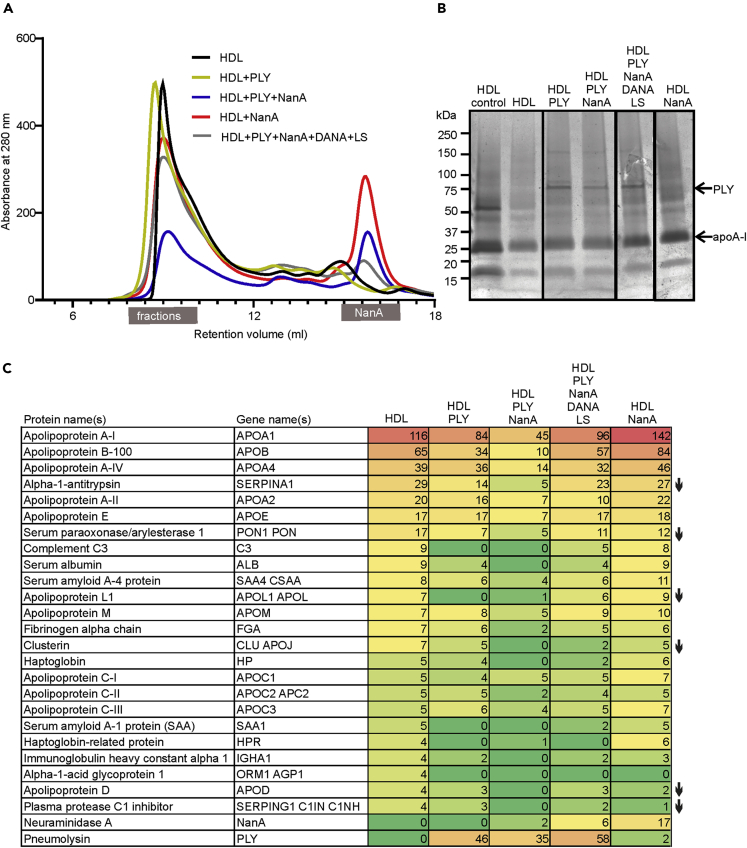
HDL modification by PLY and NanA *in vitro* (A) Size exclusion chromatography (SEC) of HDL-2 after incubation of the particles alone or in the presence of PLY, NanA, or PLY + NanA. PLY and NanA inhibitors, liposomes (LSs), and DANA were added to the control samples (HDL + PLY + NanA + DANA + LS). The main HDL-2 peak shows fractions between 7.5 and 9.5 mL retention volumes that were collected for experiments. See also Figures S2A and S2B. (B) SDS-PAGE of HDL fractions that were collected after SEC at protein concentration of 16 μg/mL. PLY is shown as a 72.8 kDa band. Major lipoprotein of HDL, ApoA-I, is shown as a 28.3-kDa band. HDL control sample showing proteins present in HDL-2 sample before SEC. (C) HDL-2 samples analyzed by MS/MS comparing the protein profile of the HDL-2 samples after SEC. See also Figure S2C and Table S2. Numbers are shown as the percentage of peptide spectrum matches (PSMs) of all identified proteins with a score of >10 or PSMs >4 in the HDL-2 sample. Reduction in protein content in PLY- and/or NanA-treated HDL samples compared to the sample without microbial molecules is marked with arrows.

To validate the relevance of PLY interaction with HDL-2 surface unesterified cholesterol and the role of NanA-mediated desialylation of HDL-2 in HDL function *in vitro*, we next analyzed the particles after incubating them with the microbial molecules. The experiment included a control sample with the inhibitors, liposomes and 2,3-didehydro-2-deoxy-N-acetylneuraminic acid (DANA) (Figure S2A), for PLY and NanA, respectively (Braun et al., 1999). The size exclusion chromatographic (SEC) profile of the HDL-2 samples showed that the major HDL-2 fractions eluted between 7.5 and 9.5 mL retention volumes, whereas NanA and PLY eluted much later at 14.5-15 mL and 20-23 mL volumes (Figures 3A and S2B). PLY bound to HDL was detected in the HDL-2 fractions (Figure 3B). The proteomic analysis of the HDL-2 samples showed that PLY was abundant on those samples that were incubated in the presence of PLY (Figure 3C and Table S2). The HDL-2 bound PLY was not functionally active, except in the sample that was incubated in the presence of the PLY and NanA inhibitors (Figure S2C). This provided further evidence for the role of HDL-2 in inhibiting PLY activity. Examination of the proteomic profiles revealed differences between the HDL-2 sample incubated with only a buffer and the PLY- and NanA-incubated samples. The PLY and/or PLY- and NanA-incubated samples showed depletion of PON-1 levels similarly as in the *ex vivo* assay. Also, reduction in alpha-1-antitrypsin, apolipoprotein L1, apolipoprotein D, complement regulator plasma protease C1 inhibitor, and clusterin was observed.

### PLY and NanA convert HDL from anti-inflammatory to pro-inflammatory and pro-atherogenic particles

**Figure 4:**
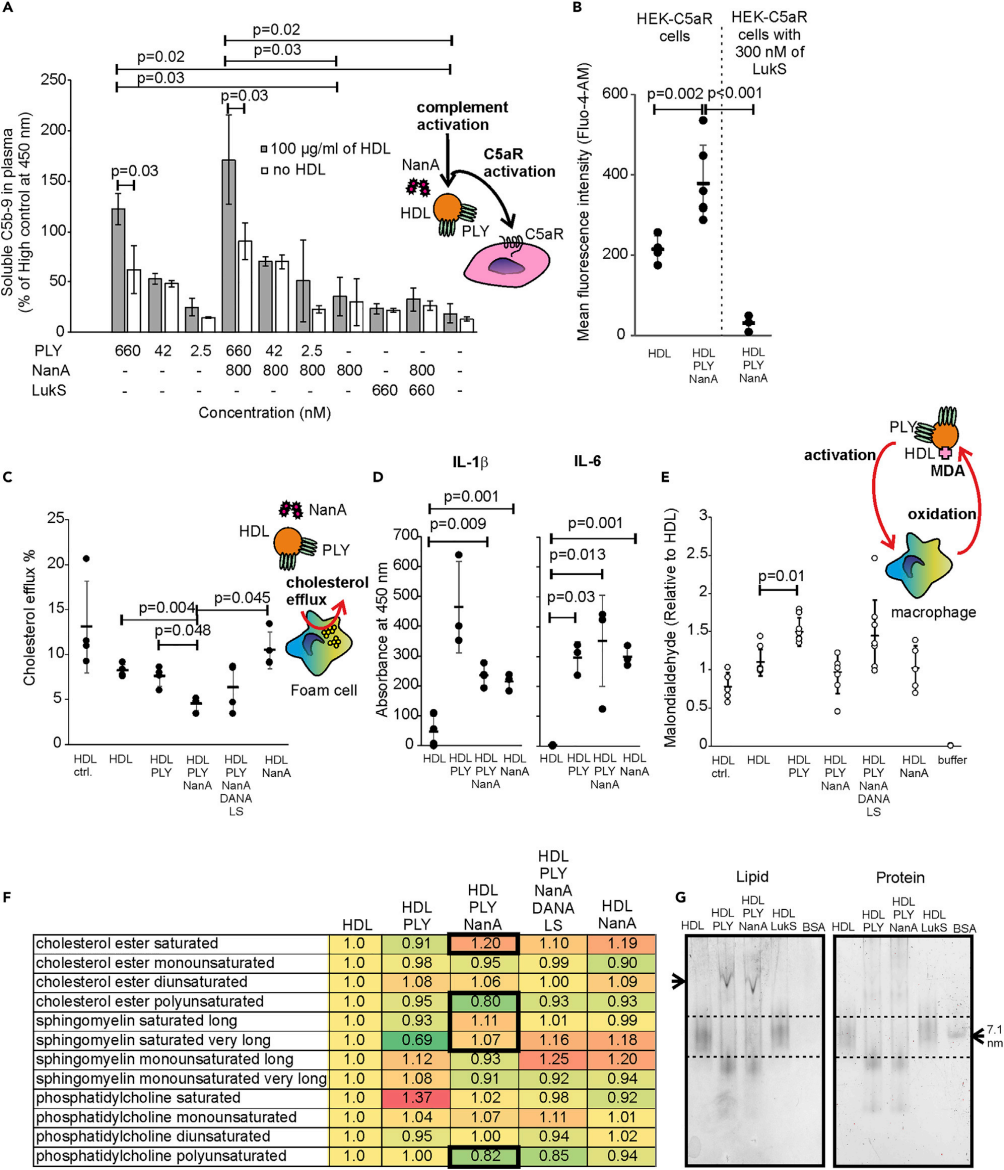
Effect of PLY and NanA on HDL function (A) Complement activation as measured by the amount of soluble C5b-9 formation in hirudin plasma with (100 μg/mL of HDL) or without (no HDL) HDL particles that were incubated in the presence or absence of PLY and/or NanA. LukS was used as a negative control for complement activation in plasma. (B) Calcium mobilization of HEK-C5aR cells in the presence of HDL-2 particles that were incubated in the presence or absence of PLY and NanA. Specificity of C5a-mediated calcium mobilization was analyzed using LukS. See also Figures S3A and S3B. (C) Cholesterol efflux from PMA-activated and cholesterol-labeled THP-1 cells in the presence of HDL-2 particles that were isolated by SEC. The samples were HDL ctrl (= HDL not run through SEC), HDL only (HDL), HDL + PLY, HDL + PLY + NanA, HDL + PLY + NanA + DANA + liposomes (LS), and HDL + NanA. Cholesterol efflux is represented as the percentage of efflux elicited in four hours. The non-specific efflux background without HDL was subtracted from the samples. (D) Release of IL-1β and IL-6 by activated THP-1 cells when exposed to HDL and PLY, NanA + PLY, and NanA-modified HDL particles. (E) The presence of MDA epitopes on HDL-2 after a four-hour incubation with activated THP-1 macrophages from the efflux assay. LS and DANA (HDL + PLY + NanA + DANA + LS) were included in the experiment to neutralize the effect of PLY and/or NanA. (F) HDL-2 samples analyzed by ESI-MS/MS to compare the lipid diversity of the HDL-2 samples after SEC. The results are expressed as fold change values obtained by dividing the molar percentage of the lipid structural category in the HDL with PLY and NanA by the corresponding values in ctrl HDL. For SM, categories long or very long included species with 15‒18 or 23‒25 acyl carbons, respectively. (G) Aggregation (arrow) of HDL particles in the presence or absence of PLY and PLY + NanA shown by lipid staining after native PAGE. LukS is used as negative control for aggregation and BSA as a size standard (7.1 nm) and negative control for lipid staining. Non-aggregated HDL is shown between the dashed lines. Statistical significant (p < 0.05) differences between samples in (A)–(E) were determined using one-way ANOVA followed by Dunnet's non-parametric multiple comparison test. Error bars showing SDs are calculated from at least three biological replicates

Due to the interactions of HDL-2 with PLY and NanA and the major modifications that were detected in HDL-2 sialylation and protein content, we next studied whether these modifications could change the anti-inflammatory role of HDL-2 to a dysfunctional and pro-inflammatory form. Modified HDL showed reduced complement resistance, as the addition of preincubated HDL-2 to hirudin plasma resulted in a significant increase in complement activation in the presence of PLY and NanA, while treatment of plasma with HDL-2 in the absence of PLY and NanA treatment did not (Figure 4A). HDL-2 preincubated with the negative control, LukS, did not increase complement activity (Haapasalo et al., 2019). Similarly, the HDL-2 that had been separated by SEC after PLY and NanA treatment showed increased complement activity and C5a formation in serum when compared to non-treated HDL-2 as detected by increase in the intracellular calcium in C5aR cells (Figure 4B) (Haapasalo et al., 2019). Pretreatment of C5aR-expressing cells with LukS, which is known to bind to C5aR and antagonize the effect of C5a (Figures S3A and S3B) (Spaan et al., 2013), completely inhibited C5aR activation indicating specific activation of C5aR cells through C5a formation induced by the PLY- and NanA-treated HDL-2. The PLY- and NanA-modified HDL particles also activated IL-6 release in human umbilical vein endothelial cells, indicating direct cell response to microbially modified HDL stimuli (Figure S3C) (Kaplanski et al., 1995). The activity of HDL-2 was tested by examining its potential to remove cholesterol from cholesterol-labeled THP-1 macrophages. Clear attenuation in cholesterol egress via PLY-NanA treated HDL2 was demonstrated. The additional effect of NanA in the reduced cholesterol efflux was expected as glycosylation has been shown to directly affect the functional properties of HDL (Sukhorukov et al., 2019) (Figure 4C). To study the ability of the modified HDL to activate inflammatory responses on cells and escape from oxidative stress, we measured release of IL-1β and IL-6 upon HDL stimuli and the presence of malondialdehyde (MDA) epitopes, the products of lipid peroxidation, on these same samples after incubation with the THP-1 macrophages (Papac-Milicevic et al., 2016). Increased amounts of IL1-β, IL-6 released in the supernatant, and MDA adducts in the HDL-2 sample incubated with PLY + NanA, PLY, or NanA indicated cell response to the bacterially modified HDL and macrophage-mediated peroxidation of the modified HDL (Figures 4D and 4E).

Further analysis of the HDL lipidome provided additional evidence for our functional data (Figure 4F) (Folch et al., 1957). The lipidomes of HDL-2 particles treated with PLY and NanA showed relative increase in saturated sphingomyelin (SM) and at the same time a decrease of polyunsaturated phosphatidylcholine (PC) species, which in turn decreases membrane fluidity (Shaikh et al., 2006). Similarity, a relative increase of saturated and a concomitant decrease of polyunsaturated species were detected in cholesterol ester (CE) species of the HDL core. Because the cholesterol uptake by HDL is compromised by decreased fluidity of the surface phospholipids (Davidson et al., 1995) and oxidative degradation may reduce the proportion of polyunsaturated lipids in the HDL, the low cellular cholesterol efflux potential and increased aggregation by PLY- and NanA-treated HDL (Figure 4G) is concordant with the lipidome changes.

## Discussion

Despite more than two decades of extensive research, no specific pathogen has been conclusively linked to atherosclerosis in humans (Lathe et al., 2014). Recent studies, however, have identified *Porphyromonas gingivalis* in the brain of patients with Alzheimer disease and in arteries of patients with cardiovascular disease, suggesting that the toxic proteases from the bacterium could be involved in development of these diseases (Dominy et al., 2019). In human clinical specimens, concentrations of cytolysins can be very high ranging from 0.33 μg/mL to 399 μg/mL (Badiou et al., 2010), and, in pneumococcal infections, these can rapidly increase through the release of PLY as result of pneumococcal autolysis (Jacques et al., 2020; Mellroth et al., 2012).

As HDL is capable of neutralizing the endotoxicity of LPS, similarly, we showed that it also inhibits the hemolytic activity of PLY (Levine et al., 1993). While LPS is carried and neutralized by a specific LPS-binding protein on HDL (Wurfel et al., 1994), the binding of PLY most likely requires lipid raft microdomains rich in unesterified cholesterol (Taylor et al., 2013). Such dynamic and small raft domains were suggested to form the surface of LDL (Hevonoja et al., 2000). Enriching HDL with PLY may require a larger total area of the raft domains, which are more likely present in the larger and more lipid-rich HDL-2 than in the smaller HDL-3 (Chapman, 1986). These raft domains would increase the membrane proportions of both cholesterol and SM having a molecular structure compatible with cholesterol (Slotte, 2016; Yetukuri et al., 2010).

Gangliosides are sialic-acid-containing glycosphingolipids found on HDL (Siegel et al., 2014). Of these, GM1 has a crucial role in protecting the host from the immune system (Cutillo et al., 2020). Herein, we demonstrate that sialic acids have a role in protecting HDL from PLY and possibly also from complement as NanA reduced HDL complement resistance. In addition, the absence of clusterin in the PLY and NanA-modified HDL correlated with the formation of soluble complement components in plasma. Clusterin is a complement regulator found on HDL. It has been suggested to inhibit formation of terminal complexes of C5b-9 and regulate lipid transport (Jenne et al., 1991). Importantly, the increase in complement activity also resulted in activation of C5aR on cells and release of IL-1β and IL-6. This indicates that the presence of PLY- and NanA-modified HDL could lead to activation of cells present in atherosclerotic intima such as endothelial cells and may thereby activate coronary plaques and trigger chronic inflammation (Oksjoki et al., 2007).

HDL has been found to contain a diverse set of enzymatically active proteins, hydroxylated derivatives of polyunsaturated fatty acids, and hydroperoxides, and although HDL is regarded as anti-oxidative and anti-inflammatory, during the acute phase reaction, oxidative and inflammatory reactions take over (Lemes et al., 2020; Navab et al., 2001; Vaisar et al., 2007). Our finding that PLY-modified HDL contains a high concentration of MDA suggests dysfunction of the particle and enhancement of oxidative reactions (Shao et al., 2010). Thus, oxidative degradation may be an additional mechanism reducing the proportion of polyunsaturated lipids in HDL-2 after treatment with PLY, NanA, or both. Once the proportion of polyunsaturated PC decreases, then the remaining oxidation-resistant saturated PCs in HDL increase, which was evident in the treated HDL-2 of this study. Since oxidative reactions are chain reactions, the HDL core lipids are also affected, seen as the reduction of polyunsaturated CE and consequent relative increase in the saturated CE. As shown here, the modifications of the HDL lipidomes by PLY and NanA have consequences for the particle aggregation susceptibility. Experiments with LDL have shown that increased levels of SM, and especially the long chain saturated species of SM, associate with increased LDL aggregation susceptibility. In contrast, increased levels of PC and especially the polyunsaturated PC species associate with a low aggregation propensity (Ruuth et al., 2018).

PON-1 and glycosylphosphatidylinositol-specific phospholipase D have been shown to protect HDL from oxidative stress (O'Brien et al., 1999; Stefanovic et al., 2010). Our observation that PLY treatment reduced levels of PON-1 in HDL-2 both *in vitro* and *ex vivo* supports previous findings that modified HDL particles are susceptible to oxidative stress (Dousset et al., 1994). The proteome profile of HDL isolated from the *ex vivo* assay also showed reduction in the presence of apolipoproteins and increase of acute phase proteins such as serum amyloid A-1 (SAA) and transthyretin. It has been shown that HDL-containing SAA loses its anti-inflammatory properties and becomes proinflammatory. Moreover, an increase of SAA in HDL leads to detachment of HDL major protein, apoA-I from the particle (Vaisar et al., 2015), which is consistent with our MS data. Detachment of apoA-I would lead to attenuated cholesterol efflux via the ABCA1 receptor on macrophages due to reduced interaction between apoA-I and ABCA1. In addition, transthyretin has been shown to exhibit protease function against apoA-I (Sharma et al., 2019).

Understanding the role of microbial molecules in promoting acute inflammation is essential. However, as shown in this study, these molecules also cause spatial and functional changes in circulating lipoproteins that may trigger the inflammatory response indirectly. Our finding of such a molecular mechanism using human HDL and the role of microbial molecules in triggering chronic inflammation in atherosclerosis could advance development of therapies to prevent this disease at a very early stage.

### Limitations of the study

Rabbits have a low HDL cholesterol profile, similar to humans, and have been used to study disease mechanisms of atherosclerosis (Yin et al., 2012). However, the human specificity of PLY observed here, and by others, indicates that the 3β-hydroxyl group of cholesterol alone does not explain PLY host tropism (Shewell et al., 2014, 2020). Notably, NanA targets the terminal sialic acid N-acetylneuraminic acid (Neu5Ac), which is abundant in human cells but not in other mammalian cells (Hentrich et al., 2016). As 90% of human HDL glycoproteins are sialylated, current transgenic animal models expressing human glycoproteins would be mainly expressing N-glycolylneuraminic acid (Neu5Gc) (Huang et al., 2014; Krishnan et al., 2017). Therefore, due to the lack of suitable animal models, this study was unable to provide explanation of PLY function *in vivo*.

## STAR★methods

### Key resources table

**Table:** 

REAGENT or RESOURCE	SOURCE	IDENTIFIER
**Antibodies**		

Anti-Pneumolysin antibody [PLY-4]	Abcam	Cat# ab71810; RRID: AB_1269828
IRDye® 800CW Donkey anti-Mouse IgG Secondary Antibody	Licor Biotechnology	Cat# 926-32212; RRID: AB_621847
Penta·His Antibody	QIAGEN	Cat# 34660; RRID: AB_2619735
Peroxidase AffiniPure Rabbit Anti-Mouse IgG (H+L)	Jackson ImmunoResearch	Cat# 315-035-045; RRID: AB_2340066

**Biological samples**		

Human plasma for *ex vivo* assays and isolation of total HDL	Hirudin anticoagulated blood from healthy individuals with written and signed consent	N/A
rabbit HDL	Dr. Matti Jauhiainen; this study	N/A
HDL-2	Dr. Matti Jauhiainen; this study	N/A
HDL-3	Dr. Matti Jauhiainen; this study	N/A

**Chemicals, peptides, and recombinant proteins**		

Recombinant *Streptococcus pneumoniae* serotype 2 Pneumolysin (PLY)	Cusabio	Cat# CSB-EP312102FNF-200
Neuraminidase (NanA)	Dr. Samantha J King; this study	SP_1693 (aa303-777); Syed et al., 2019
Maackia Amurensis Lectin II (MAL II), Unconjugated	Vector Labs	Cat# L-1260-2
Bovine serum albumin (BSA) Fatty acids free lyophilized	Biowest; Biotop	Cat# P6156
Phorbol 12-myristate 13-acetate (PMA)	Sigma	Cat# P8139
Saponin, pract., from Quillaja *Saponaria Molina*	Acros Organics	Cat# 10157800
o-phenylenediamine dihydrochloride, OPD tablets 2mg	Dako	Cat# S2045
Fetal bovine Serum	Gibco	Cat# A3840402
Penicillin/Streptomycin (10,000 U/mL)	Gibco; Lonza	Cat# 15140122
Albumin from human serum	Sigma	Cat# A3782
3'-Sialyllactose sodium salt	Carbosynth	Cat# OS04397
6'-Sialyllactose sodium salt	Carbosynth	Cat# OS04398
N-Acetyl-2,3-dehydro-2-deoxyneuraminic acid	Carbosynth	Cat# MA06939
4-Methylumbelliferyl N-acetyl-a-D-neuraminic acid sodium salt	Carbosynth	Cat# EM05195
16% Formaldehyde (w/v), Methanol-free	Pierce	Cat# 28906
Probenecid	Sigma	Cat# P8761
18:0 PC (DSPC) 1,2-distearoyl-sn-glycero-3-phosphocholine	Avanti Polar Lipids	Cat# 850365
Ganglioside GM₁, Ammonium Salt, Bovine Brain	Merck	Cat# 345724
Cholesterol (ovine wool, >98%)	Avanti polar Lipids	Cat# 700000
Chloroform, 99+%, extra pure	Acros organics	Cat# AC158210010
Bromophenol blue	Merck	Cat# 8122
DAPI (4',6-Diamidino-2-Phenylindole, Dilactate)	Invitrogen	Cat# D3571
Fluo-4 AM, Fluorescent labeling reagent	Abcam	Cat# ab241082
Sudan black	Merck	Cat# 1387
HBSS	Gibco	Cat# 14175046
EndoGRO™-LS complete media kit	Merck	Cat# SCME001
Glutamax	Gibco	Cat# 35050038
Hepes buffer	Gibco	Cat# 15630056
Hirudin blood tube	Roche Diagnostics	Cat# 06675751
BD Vacutainer 6 mL PET tube for Haematology (K₂EDTA)	BD Vacutainer	Cat# 367864A
4–20% Mini-PROTEAN® TGX™ Precast Protein Gels	BioRad	Cat# 4561094
4–20% Mini-PROTEAN® TGX Stain-Free™ Protein Gels	BioRad	Cat# 4568094
Monolith NT.115 Premium Capillaries	Nanotemper	Cat# MO-K025

**Critical commercial assays**		

Superdex 200 10/300 GL	GE Healthcare	Cat# 15182085
Avanti Extruder Set with Holder/Heating Block	Avanti polar; Sigma-Aldrich	Cat# 610000-1EA
iBlot™ 2 Transfer Stacks, nitrocellulose, regular size	Invitrogen; Thermo Fisher Scientific	Cat# IB23001
MicroVue SC5b-9 (TCC) Plus Enzyme Immunoassay	Quidel	Cat# A020
Cholestrol efflux assay kit (Cell-based)	Abcam	Cat# ab196985
Oxidized HDL Assay Kit (MDA-HDL, Human)	Abcam	Cat# ab242308
Protein Labeling Kit RED-NHS 2nd Generation	Nanotemper	Cat# MO-L011
Human IL-1β DuoSet ELISA	R&D Systems	Cat# DYI201
Human IL-6 DuoSet ELISA	R&D Systems	Cat# DY206
Human TNF-alpha DuoSet ELISA	R&D Systems	Cat# DY210

**Experimental models/cell lines**		

HEK293T-C5aR, HEK 293T cells expressing G protein Gα16 and C5aR	Haapasalo et al. 2019	Acc.(NM_00173)
HUVEC cells	Dr. Mikko Helenius	N/A
THP-1 cells	ATCC	TIB-202™

**Oligonucleotides**		

Neuraminidase (NanA)	Dr. Samantha J King; Syed et al., 2019	SP_1693
Primer: SP_1693 (aa303-777) pOPINF Forward: AAGTTCTGTTTCAGGGCC CGCCTGAAGGAGCGGCTTTAAC	Syed et al., 2019	SP_1693
Primer: SP_1693 (aa303-777) pOPINF Reverse: ATGGTCTAGAAAGCTTTA ATTTTTGCTCAAAAATTCCCA	Syed et al., 2019	SP_1693
Primer: 3′LukS-K281C(NotI) Reverse: ATATGCGGCCGCTCAATTATGTC CTTTCACGCAAATTTCATGAGTT TTCC	Haapasalo et al., 2018	LukS (Q53746_STAAU)
Primer: 5′LukS-Y113H Forward: GTCAAACATTAGGTCATAAC ATAGGTGGTAATTTTAATAG	Haapasalo et al., 2018	LukS (Q53746_STAAU)
Primer: 3′LukS-Y113H Reverse: TTACCACCTATGTTATGACCTA ATGTTTGACTAAC	Haapasalo et al., 2018	LukS (Q53746_STAAU)
Primer: 5′LukS(BamHI) Forward: CGGGATCCAAAGCTGATAAC AATATTGAG	Haapasalo et al., 2018	LukS (Q53746_STAAU)

**Software and algorithms**		

FlowJo v10.1r5 software	FlowJo	https://www.flowjo.com/solutions/flowjo/downloads
MassHunter Workstation qualitative analysis software, version B.08.00	Agilent Technologies	https://www.agilent.com/en/products/software-informatics/masshunter-suite/masshunter-qualitative-analysis-gcms
Lipid Mass Spectrum Analysis (LIMSA) Software, version 1.0	Haimi et al., 2006	N/A
SPSS package software version 24	IBM	https://www.ibm.com/support/pages/downloading-ibm-spss-statistics-24
SEQUEST software included with proteome discoverer package	Yates Laboratory	http://proteomicswiki.com/wiki/index.php/SEQUEST
Proteome Discoverer™ Software version 1.4	Thermo Scientific	https://www.thermofisher.com/order/catalog/product/OPTON-30810#/OPTON-30810
Image Lab software, version 6.01, built 34	Bio-Rad	https://www.bio-rad.com/en-fi/product/image-lab-software?ID=KRE6P5E8Z
NT Control Software	Nanotemper	provided in package for Monolith NT.115 pico machine
MO affinity control analysis version 2.3	Nanotemper	provided in package for Monolith NT.115 pico machine

### Resource availability

#### Lead contact

Further information and requests for resources and reagents should be directed to and will be fulfilled by the lead contact, Dr. Karita Haapasalo (karita.haapasalo@helsinki.fi).

#### Materials availability

Materials used or generated in this study will be available upon reasonable request.

#### Data and code availability

The published article includes all data generated or analyzed during this study.

### Experimental model and subject details

#### HDL isolation

HDL samples were prepared from freshly isolated plasma of normolipidemic healthy volunteers obtained from the Finnish Red Cross Blood Service by sequential flotation ultracentrifugation using potassium-bromide for density adjustment as previously described using a reference method from (Havel et al., 1955). The volunteers included both genders between ages 25-60 years old. The methodology and the quality control of the preparations were as follows: Plasma VLDL (d < 1.006 g/mL), LDL (1.019 < d < 1.063 g/mL), total HDL (1.063< d < 1.210 g/mL), HDL-2 (1.063 < d < 1.125 g/mL) and HDL-3 (1.125 < d < 1.210 g/mL). All steps were done in KBr-salt solution to avoid any artificial chemical modifications. The purity of the HDL-2 fraction was analyzed with regard to apoB-100 using human apoB-100 ELISA with apoB-100 detection sensitivity of about 6 ng/ml (Abcam, Cambridge, UK). The isolated HDL-2 was practically devoid of apoB-100 as the concentration of apoB-100 in the preparation was 8 ng/ml. The measured total protein and lipid composition of the isolated HDL-2 (as mass%) were as follows: protein 41%, phospholipids 27%, cholesterol esters 24%, free cholesterol 5% and triglycerides 3%. Isolated HDL-2 was extensively dialyzed against phosphate buffered saline (PBS pH 7.4) before analysis (Havel et al., 1955).

#### Isolation of plasma and red blood cells

Blood was drawn into hirudin tubes (Roche Diagnostics, Mannheim, Germany) from healthy human volunteers after informed written and signed consent (Ethical Committee decision HUS/135/2020, Hospital district of Helsinki and Uusimaa). The volunteers included both genders between ages 25-60 years old. To separate the plasma from blood cells the sample was centrifuged at 300 × g for 10 min at room temperature. Collected cells were washed twice with phosphate buffer saline (PBS pH 7.4) and 1% red blood cell suspension was prepared in PBS.

### Method details

#### Hemolysis assay

The isolated red blood cells were incubated in round bottom 96-well polystyrene plates (Nunc, Roskilde, Denmark) in PBS with different concentrations of recombinant pneumolysin (PLY) (Cusabio technology LLC, Houston, TX) and in the presence or absence of 800 nM of a recombinantly expressed *S. pneumoniae* NanA neuraminidase fragment containing the functional C-terminal domain (amino acids 330-777) (Syed et al., 2019). Incubation was done in an orbital rotator for 30 min at 37 °C. Red blood cells were pelleted by centrifugation at 300 × g for 10 min, and 30 μl of the supernatant was diluted with 70 μl PBS. Hemolysis was detected by measuring the absorbance at 405 nm with iEMS Reader MF (Labsystems Diagnostics Oy, Vantaa, Finland). Inhibition of lysis by HDL was measured by incubating increasing concentrations of HDL-2 (0-100 μg/ml) with 10 nM of PLY and 1% red blood cells in PBS with or without 800 nM of NanA in round bottom 96-well polystyrene plates (Nunc). Hemolysis was measured at 405 nm as described above. Increasing concentrations of Saponin (1.52 × 10^-5^% to 0.1%, Acros Organics, Fair Lawn, NJ) in Roswell Park Memorial Institute (RPMI) 1640 medium (Gibco) were used as a positive control for hemolysis, while liposomes were used to analyze the role of cholesterol in PLY inhibition.

#### Permeability assay

THP-1 monocyte-derived macrophages obtained from American Type Culture Collection (ATCC, Virginia, USA) were cultured in RPMI medium supplemented with penicillin, streptomycin, Glutamax and 10% (v/v) fetal bovine serum at 37 °C in the presence of 5% CO_2_. Cells were centrifuged at 300× g for 10 min at 4 °C. The supernatant was removed, and the pellet was resuspended in RPMI medium supplemented with 0.05% human serum albumin (Sigma Aldrich, St. Louis, MO). Cells were used at a concentration of 1 x 10^6^ cells/ml. The nucleus staining dye 4′,6-diamidino-2-phenylindole (DAPI) (Invitrogen, Carlsbad, CA) was added to the cells (1 μg/ml). HDL particles (suspended in PBS pH 7.4) were prepared as mentioned before. THP-1 cells were incubated with HDL (0-100 μg/ml) in the presence of PLY (100 nM) (Cusabio), in the presence or absence of the recombinant NanA (800 nM) in 96 well round clear-bottom plates (Nunc) at 37 °C for 30 min with mild shaking. 100 nM of *Staphylococcus aureus* toxin component LukS was included as a negative control (Haapasalo et al., 2019). Incubated cells were washed with RPMI medium with fatty acid free 0.05% human serum albumin (Sigma) (RPMI-HSA) medium at 300 × g for 10 min at room temperature. The supernatant was discarded, and cells were resuspended in 1% paraformaldehyde (v/v) in RPMI-HSA medium. Events were acquired on BD LSR Fortessa flow cytometer (laser 405 nm filter 450/40). The signal threshold for cell permeability was set by measuring fluorescence of cells incubated with DAPI alone, and the frequency of permeable cells compared to the parent population was determined using FlowJo 10.1r5 (FlowJo LLC, Ashland, Oregon).

#### Preparation of liposomes

Liposomes were prepared using 1,2-distearoyl-sn-glycero-3-phosphocholine (DSPC) and unesterified cholesterol at a 7:3 ratio and following thin film protocol (Briuglia et al., 2015; Jimah et al., 2017). Briefly, to formulate 1 mM liposomes, 700 μM of DSPC and 300 μM of cholesterol were prepared by dissolving appropriate amounts in 2 ml chloroform in a round bottom flask. The mixture was then evaporated in a rotary evaporator for 30 min at 35°C to obtain a dry lipid film. This film was further dried in a vacuum oven for 1 h at 40 °C. After overnight incubation, the liposome mixture was sonicated using probe sonicator for 3 min at intervals. The multilamellar vesicles were then extruded at 90 °C with polycarbonate filters (100 nm pore size) using Avanti Polar Lipid extruder kit (Sigma-Aldrich, St. Louis, MO) to obtain unilamellar vesicles. Finally, the vesicles were concentrated, and the concentrated liposome solution was washed with ultrapure water and centrifuged (3 times) until a clear solution was collected in the filtration tube. Then, 20 μl of the liposome concentrate was added to 700 μl of PBS in a cuvette and the hydrodynamic size was measured using dynamic light scattering (DLS) instrument. The purified liposomes displayed a hydrodynamic size of 172 nm with a polydispersity index (PDI) of 0.08.

#### Binding of PLY to GM1 coated HDL

To analyze binding of PLY and NanA to HDL-2, 80 μg/ml of HDL particles were coated on 96-well plates (SpectraPlate-96 HB, PerkinElmer, Waltham, MA) in bicarbonate buffer (pH 9.6) overnight at 4 °C. Fatty acid free bovine serum albumin (Biowest, Rue du Vieux Bourg, Nuaillé, France) (BSA) coated wells were used to control nonspecific binding of the proteins to the wells. Wells were washed once with PBS where after the coated plates were incubated with increasing concentrations of PLY and NanA at 37 °C for 1 h. Wells were washed with PBS three times and blocked with 3% BSA in PBS for 2 h at 37 °C. After one wash with PBS, the wells were incubated with 0.4 μg/ml penta-His antibody (Qiagen, Hilden, Germany) in PBS with 0.05% BSA (BSA/PBS) at 37 °C 1 h. Wells were washed three times with PBS and incubated with 1:2000 dilution of HRP conjugated anti-mouse IgG antibody (Jackson ImmunoResearch Laboratories, West Grove, PA) in 0.05% BSA/PBS at 37 °C for 1 h. After three washes with PBS, *o*-phenylenediamine dihydrochloride (OPD) substrate (Dako Corporation, Carpenteria, CA) was added according to manufacturer instructions. The reaction was stopped with 0.5M H_2_SO_4_ and absorbance was measured at 492 nm. Binding was calculated after subtracting the background absorbance of the BSA background control.

To analyze binding of PLY to different HDL samples (human HDL, rabbit HDL, HDL-2 and HDL-3), 80 μg/ml of each HDL sample was coated on 96-well plates (SpectraPlate-96 HB) in bicarbonate buffer (pH 9.6) overnight at 4 °C. BSA coated wells were used to control nonspecific binding of the proteins to the wells. Wells were washed once with PBS and blocked with 3% fatty acid free BSA (Biowest) in PBS for 2 h at room temperature. Wells were washed once with PBS and then incubated with increasing concentrations of PLY at 37 °C for 1 h. After three washes with PBS, the wells were incubated with 1 μg/ml of rabbit anti-PLY antibody (Abcam) in 0.05% BSA/PBS at 37 °C 1 h. Wells were washed three times with PBS and incubated with 1:2000 dilution of HRP conjugated anti-rabbit IgG antibody (Jackson ImmunoResearch Laboratories, West Grove, PA) in 0.05% BSA/PBS at 37 °C for 1 h. After three washes with PBS, OPD substrate (Thermo Fischer Scientific) was added according to manufacturer’s instructions. The reaction was stopped with 0.5M H_2_SO_4_, and absorbance was measured at 492 nm.

Because of the important anti-inflammatory role of monosialotetrahexosylganglioside (GM1) and the high affinity between lipoproteins and ganglioside hydrophobic ceramide moiety (Rebbaa and Portoukalian, 1995), HDL particles were coated with GM1 to study the interaction of sialylated carbohydrates with microbial molecules. HDL-2 particles were incubated with increasing concentrations of GM1 (0-10 μg/ml) for 1 h at 37 °C and washed three times with PBS. Next, the wells were incubated with 30 nM of PLY or NanA for 1 h at 37 °C and washed three times with PBS followed by blocking with 3% BSA/PBS as described above. To study the effect of sialic acid removal by NanA in PLY binding to HDL the HDL-2 coated wells were washed once with PBS, and incubated with or without 10 μg/ml of GM1 for 1 h at 37 °C. After three washes with PBS, wells were incubated with increasing concentrations of NanA (0-800 nM) for 1 h at 37 °C. Next, wells were incubated with 30 nM of PLY or PBS for 1 h at 37 °C, and washed again three times with PBS before blocking, incubation with antibodies and detection as described above.

#### Removal of sialic acids from HDL by NanA

To detect removal of sialic acids from HDL, Maackia Amurensis Lectin II (MAL-II) (Vector Labs, Burlingame, CA) was labeled with NT-647 dye (NT647-MALII) following the manufacturer instructions (NanoTemper Technologies GmbH, München, Germany). NT647-MAL-II was diluted in microscale thermophoresis (MST) buffer (25 mM HEPES, gibco; 150 mM KCl; 0.01% NaN_3_; 0.01% Tween-20) and was centrifuged (16 000 × g, 5 min, 4 °C) to remove any aggregates. HDL-2 was diluted (1:1) in PBS in a 16 step dilution series and constant concentration of labeled NT647-MAL-II was added to the samples. Finally, samples were transferred to premium capillaries (NanoTemper) for MST measurement using Monolith NT.115Pico (NanoTemper) instrument. EC_50_ values were obtained from the MST data using the Hill equation in MO.Affinity Analysis software (NanoTemper).

Release of terminal sialic acids from HDL surfaces was detected using modified thiobarbituric acid (TBA) assay. The HDL (470 μg/ml) particles were incubated with NanA for 30 min at 37 °C in PBS. The chromophore was developed, and free sialic acids were quantified relative to the absorbance of the chromophore at 550 nm, following the previously published protocol (Warren, 1959).

#### Neuraminidase activity assay

To analyze the effect of the sialic acid analogs on NanA activity, 4-Methylumbelliferyl N-acetyl-α-D-neuraminic acid (MUAN) (Carbosynth) fluorescence substrate was used, and the assay was modified from the previously published method (Potier et al., 1979; Syed et al., 2019). NanA (5 nM) was incubated with MUAN (0.003%) in sodium acetate buffer (pH 7) for 5 min at 37 °C in 96-well black clear-bottom microplates (Perkin-Elmer, Waltham, MA, US) in the presence of increasing concentrations (0-800 nM) of 3′-Sialyllactose (3SL) (Carbosynth, Compton, Berkshire, UK) or 2,3-didehydro-2-deoxy-N-acetylneuraminic acid (DANA) (Carbosynth). NanA activity was checked by measuring the release of the fluorophore by measuring 355 nm (excitation) and 460 nm (emission) in the Hidex Sense microplate reader (Hidex, Turku, Finland).

#### Size exclusion chromatography (SEC)

HDL particles (1.25 mg/ml) were incubated in the presence or absence of NanA (10 μM), in the presence or absence of PLY (1.7 μM), and in the presence or absence of liposomes and DANA (20 μM) (Carbosynth), in LoBind tubes (Eppendorf, Hamburg, Germany) for 20 min at room temperature. Next, the samples were run through SEC column (Superdex 200 10/300 GL column Global Life Sciences Solutions LLC, Marlborough, MA, United States) at 0.5 ml/min flow rate in PBS. Fractions of 300 μl were collected, and the HDL-2 fractions from 8.3-10.0 ml were pooled and stored immediately at -80 °C until further analysis. The peaks from SEC were run on Mini-PROTEAN TGX Stain-Free precast gels 4-20% in non-reducing conditions (Bio-Rad Laboratories, Hercules, CA) at 120 V, 60 min, stained with silver stain and imaged with Gel Doc XR+ system using silver stain protocol in Image Lab software (Bio-Rad). Protein concentrations of the samples were measured with the Bicinchoninic acid assay (BCA) (Pierce Biotechnology, Rockford, Il, US) according to manufacturer instructions. To estimate the amount of HDL in the assays, the concentrations of HDL samples are expressed as the equivalent concentration (μg/ml) or weight (μg) of protein.

#### Mass spectrometric analysis for protein composition

The proteomic analyses were performed at Helsinki University proteomics unit. A total of 5 μg of each isolated HDL-2 fraction was purified by reversed-phase chromatography columns (C18 material, eluted with 50% CH_3_CN, 0.1% TFA) (PMID: 23602568). The dried peptides were reconstituted (1% CH_3_CN, 0.1% TFA), and the MS analysis was performed on an Orbitrap Elite ETD mass spectrometer (Thermo Fischer Scientific) using Xcalibur Version 2.7.1 coupled to a Thermo Scientific nLCII nanoflow HPLC system. Peak extraction and subsequent protein identification were achieved using Proteome Discoverer software (Thermo Fischer Scientific). Calibrated peak files were searched against the human component of the UniProt database by the SEQUEST search engine. Error tolerances on the precursor and fragment ions were ±15 ppm and ±0.8 Da, respectively. For peptide identification, a stringent cut-off (1% false discovery rate) was used.

#### *Ex vivo* binding assay

Hirudin anticoagulated blood was incubated in the presence or absence of 200 nM PLY or 200 nM PLY and 1.2 μM NanA for 1 h at 37 °C in a rotator. Plasma was separated from blood by centrifugation (10 min 1,000 × g) and HDL samples were prepared by sequential flotation ultracentrifugation using potassium-bromide for density adjustment as described above. The HDL proteomes were analyzed by MS/MS and western blotting (WB). For WB 40 μl of the samples and 50 ng of PLY were run on SDS-PAGE using Mini-PROTEAN TGX Stain-Free precast gels (Bio-Rad) under non-reducing conditions for 75 min at 150 V. The proteins were transferred (iBlot, Thermo Fisher Scientific) to nitrocellulose membrane (iBlot™ 2 Transfer Stack, Invitrogen) and blocked with 3% milk/PBS. The membrane was then incubated with 0.4 μg/ml of mouse anti-penta-His antibody (Qiagen, Hilden, Germany) in 0.3% milk/PBS for 1 h at 37 °C and after washing with PBS with 1:5000 dilution of IRDye® 800CW Donkey anti-mouse IgG Secondary Antibody (LI-COR) for 1 h at 37 °C in 0.3% milk-PBS. The membrane was washed with PBS and imaged using Odyssey® CLx Imaging System (LI-COR).

#### Complement activation on HDL particles

HDL-2 particles were incubated in the presence or absence of NanA (800 nM) and in the presence of PLY (2.5, 42 or 660 nM) for 30 min at 37 °C. Controls without HDL, incubated with NanA (800 nM) and PLY (2.5, 42 or 660 nM) were included to measure the background complement activation. *S. aureus* toxin LukS (660 nM) in the presence and absence of NanA fragment (800 nM) was included as a negative control (Haapasalo et al., 2019). Next, 1:5 volume of 50% hirudin plasma diluted in PBS was added to the samples and incubated for 30 min at 37 °C. Complement activation was stopped immediately by adding 10 mM EDTA. Samples were stored at -20 °C until further use. Complement activation was measured by quantifying the formation of the soluble C5b-9 (sC5b-9) using a Quidel ELISA kit (San Diego, CA). Samples were diluted 1:40 in the provided dilution buffer, and manufacturer instructions were followed.

#### Calcium mobilization assay

Preparation of Human Embryonic Kidney (HEK) 293T cells (Invitrogen), stably expressing G protein Gα16 and human C5aR has been described before (Haapasalo et al., 2019). HEK-C5aR cells (5 x 10^6^ cells/ml) were loaded with 2 μM Fluo-4-Am ester (Abcam) in HBSS media (Gibco) in the presence of 0.05% human serum albumin (Sigma), 10 mM HEPES (Gibco) and 2.5 mM Probenecid (Sigma) for one hour at 37 °C, gently agitating and protected from light. Next, cells were washed with the same buffer and diluted to 5 x 10^5^ cells/ml with or without 300 nM of LukS antagonist. The PLY and NanA or buffer treated and SEC isolated HDL samples were incubated in 10% normal human serum for 30 min at 37 ^o^C at a protein concentration of 17 μg/ml and placed immediately on ice before analysis. C5aR activity was measured by running the cells (2.5 x 10^5^ cells /ml) in flow cytometry for 30 seconds without stimulus and 120 seconds after adding the stimulus; C5a (Abcam) or diluted HDL sample incubated with 10% serum (Figure S3A). C5aR activity was calculated by subtracting the mean fluorescence intensity of cells before stimulus from mean fluorescence intensity of cells after the stimulus.

#### Cholesterol efflux assay

THP-1 cells (100 000 cells/well in a 96-well plate) were activated with 100 nM Phorbol 12-myristate 13-acetate (PMA) (Sigma-Aldrich, St. Louis, MO) in RPMI medium for 72 h and cultured at 37 °C in 5% CO_2_ atmosphere. Cholesterol efflux was determined using a cell-based Cholesterol Efflux Assay kit (Abcam, Cambridge, UK) according to manufacturer’s instructions. Activated cells were labeled with fluorescent cholesterol for 16 h and washed with the RPMI-HSA medium. A total of 5 μg protein weight of the treated HDL samples were added to the culture of cholesterol-labeled cells. The HDL treated samples were 1) HDL only, 2) HDL + PLY, 3) HDL + PLY + NanA, 4) HDL + PLY + NanA + DANA + liposomes and 5) HDL + NanA. These were prepared as described in SEC section: HDL particles (1.25 mg/ml) were incubated with NanA (10 μM), PLY (1.7 μM), liposomes and DANA (20 mM) for 20 minutes at room temperature. Specific inhibitors of PLY and NanA, liposomes (LS) and DANA (HDL+PLY+NanA+DANA+LS) were included into the experiment to neutralize the effect of PLY and NanA. The Cholesterol efflux of HDL (HDL ctrl.) that was not run through SEC was also measured. Incubation of the HDL treated samples with cholesterol labeled cells was done for four hours at 37 °C with 5% CO_2_. Fluorescence was measured for media (containing the HDL) and the solubilized donor cells at 485 nm (excitation) and 520 nm (emission) in the Hidex Sense microplate reader (Turku, Finland). Total cellular efflux mediated by HDL was calculated by the ratio between fluorescence intensity of media and fluorescence intensity of cell lysate plus media after subtraction of non-specific efflux background without HDL (average+/-SD being 16.1+/-1.5%) and presented as %.

#### Measuring cellular release of cytokines

THP-1 monocyte-derived macrophages (ATCC) were cultured in RPMI medium supplemented with penicillin, streptomycin, 25 mM HEPES, 2 mM Glutamax and 10% (v/v) fetal bovine serum. Human umbilical vein endothelial cells (HUVECs, a kind gift from Mikko Helenius, University of Helsinki, Helsinki, Finland) were grown using EndoGRO-LS complete media kit according to manufacturer’s instruction (SCME001 from Merck-Millipore) as confluent monolayers at density of 150 000 cells/well in 24-well tissue culture plates (Greiner) at 37 °C in the presence of 5% CO_2_. THP-1 cells (400 000 cells/well) were differentiated using 150 nM PMA (Sigma-Aldrich, St. Louis, MO) for 72 hours in 24-well tissue culture plates. Differentiated THP-1 cells were cultured in fresh media overnight. Cells were kept in serum free media for 20 minutes before samples were added. To measure the cellular release of IL-1β and IL-6 by both cell types a total of 0.94 μg of the treated HDL samples were added to the cells in 250 μl volume of serum free culture media and were incubated for 20 hours. The incubation media was collected, and the concentrations of IL-1β and IL-6 were measured by ELISA assays (Human IL-1β DuoSet ELISA, cat. DY201; Human IL-6 DuoSet ELISA, cat. DY206; and Human TNF-alpha DuoSet ELISA cat. DY210) following the manufacturer’s instructions (R&D Systems).

#### Measuring Malondialdehyde (MDA) adducts on HDL

As an indicative biomarker of the oxidative stress, we assessed the presence of malondialdehyde (MDA) adducts on HDL. The supernatant from activated THP-1 macrophages that had been incubated with fluorescently labeled cholesterol was stored at -20 °C in 96 well round bottom plates (Nunc) until further use. The MDA epitopes were detected on the HDL suspended in this supernatant using a commercial Oxidized HDL Assay Kit (MDA-HDL, Human) from Abcam according to manufacturer’s instructions. This ELISA kit captures MDA epitope containing particles and detects ApoA-I from samples.

#### Mass spectrometry analyses of HDL lipid composition

Total lipids of the isolated HDL particles were extracted for lipid mass spectrometry (MS) with the method previously described (Folch et al., 1957). Aliquots of the lipid extracts were dissolved in chloroform/methanol (1:2 v/v) and spiked with a mixture of internal lipid standards containing representatives for all the lipid classes analyzed. Just prior to MS, NH_4_OH was added to aliquots of the sample extracts to give 2% solution, which supported ionization and prevented sodium adduct formation. The samples were introduced via a syringe pump into the electrospray ionization (ESI) source of a triple quadrupole MS (Agilent 6410 Triple Quad LC/MS; Agilent Technologies, Inc., Santa Clara, USA) at a flow rate of 10 μl/min. MS/MS precursor ion scans of m/z 184 and m/z 369 were used to detect phosphorylcholine-containing phospholipid species (PC and SM) and CE species, respectively. The ESI-MS/MS instrument was set to a source temperature of 250 °C and collision energies optimized for each lipid class (13-30 eV) were used. Nitrogen was used as the collision, nebulizing (40 psi), and drying gas (3 l/min). Data analysis of the mass spectra was performed by using MassHunter Workstation qualitative analysis software (Agilent Technologies, Inc.), and the individual lipid species were quantified and converted to molar percent data using the internal standards and Lipid Mass Spectrum Analysis (LIMSA) software, which has an inbuilt deisotoping routine that will automatically correct for an overlap of isotope peaks (Haimi et al., 2006). The results are expressed as fold change values obtained by dividing the molar% of the lipid structural category in the HDL with PLY and NanA by the corresponding values in untreated HDL.

#### Native PAGE and lipid staining

Blood was collected in EDTA tubes (BD Vacutainer) and HDL was isolated from EDTA anticoagulated serum as described above. HDL in a protein concentration of 100 μg/ml was incubated in PBS (pH 7.4) in the presence of 1.4 μM pneumolysin (Cucabio) and/or in presence of 1 μM NanA, or 1.4 μM LukS toxin in 96-well round bottom plates at 37 °C for 1 hour. 2 x native sample buffer (62.5 mM Tris-HCl, pH 6.8, 40% glycerol, 0.01% bromophenol blue) was added in a 1:1 ratio to the samples. A total of 5 μg of HDL protein content in the presence or absence of 1.2 μg of LukS, or 3 μg of pneumolysin in the presence or absence of 1.4 μg of NanA were run on Mini-PROTEAN TGX Stain-Free precast gels (Bio-Rad). 15 μg BSA (fatty acid free) (Biotop, Turku, Finland) was run on the same gel as a 7.1 nm size standard and as a negative control for specific labeling of lipids. Gel was run for 1 hour at 100 V in 25 mM Tris, 192 mM glycine buffer, pH 8.3, and imaged with Gel Doc XR+ system using stain-free gel protocol in Image Lab software (Bio-Rad). To detect the lipids, the gel was stained with the Sudan Black B (Merck) dye overnight at room temperature. Briefly, 500 μg of Sudan Black B was dissolved in 20% acetone, 12.5% acetic acid dissolved in ddH_2_O. The solution was stirred for 30 min at room temperature and added to the gel and incubated overnight at room temperature. The following day the Sudan Black B staining solution was removed, and the gel was destained by incubating with the destaining solution (15% acetic acid, 20% acetone dissolved in ddH2O) for 15 min. The procedure was repeated three times before the gel was imaged with Gel Doc XR+ system using Coomassie blue stain protocol in Image Lab software.

### Quantification and statistical analysis

Experiments were performed using at least three biological replicates. For statistical analyses SPSS package was used (SPSS version 24 IBM Statistics). First, Kolmogorov-Smirnov normality test was used to analyze whether variables were normally distributed. Statistical significance between two samples was analyzed using Mann-Whitney U test. For multiple comparisons and samples with unequal variances, one-way ANOVA supplemented with Dunnett’s post hoc test was used (SPSS version 24 IBM Statistics). Standard p-value threshold of <0.05 was used to indicate statistical significance.

## Supplemental information

Document S1. Figures S1–S3 and Table S1

Table S2. Protein identification of HDL-2 samples after gel filtration chromatography, related to Figure 3C
