# Supplementary material for: Streptococcus pneumoniae pneumolysin and neuraminidase A convert high-density lipoproteins into pro-atherogenic particles
Source: iScience. 2021 May 12;24(6):102535. doi: 10.1016/j.isci.2021.102535 (PMC8175417; doi:10.1016/j.isci.2021.102535)
Supplement: Document S1. Figures S1–S3 and Table S1 [file mmc1.pdf]

iScience, Volume *24*

Supplemental information

Streptococcus pneumoniae pneumolysin and neuraminidase A convert high-density lipoproteins into pro-atherogenic particles

Shahan Syed, Eija Nissilä, Hanna Ruhanen, Satoshi Fudo, Meztlli O. Gaytán, Sanna P. Sihvo, Martina B. Lorey, Jari Metso, Katariina Öörni, Samantha J. King, Oommen P. Oommen, Matti Jauhiainen, Seppo Meri, Reijo Käkelä, and Karita Haapasalo

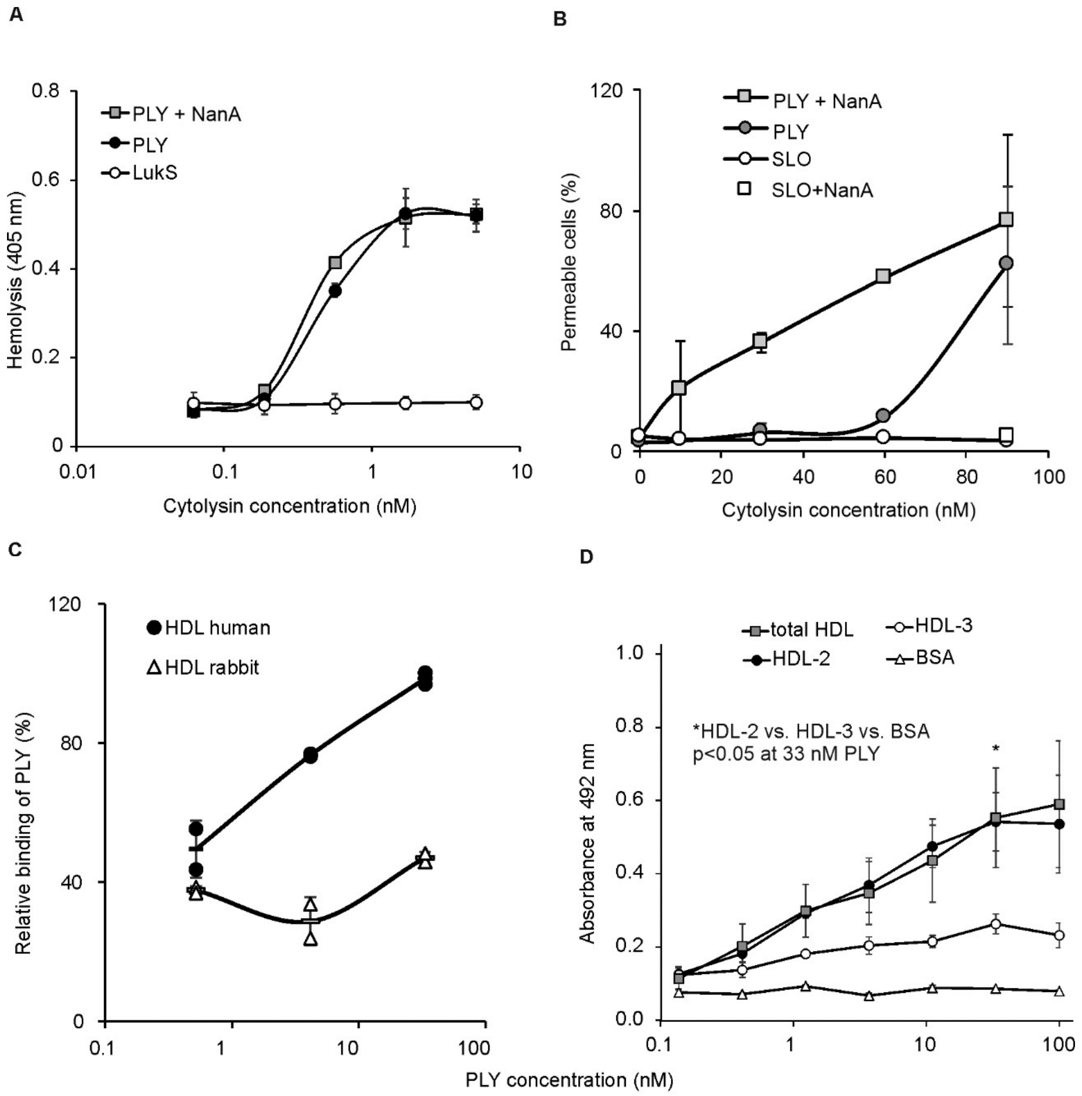

Figure S1. Lysis of red blood cells and cultured monocytic cells by PLY and NanA and binding of PLY to different HDL particles (Related to Figure 1). A, Red blood cells isolated from hirudin anticoagulated blood were incubated with increasing concentrations of (x-axis) cytolysins PLY or LukS negative control in the presence or absence of NanA (800 nM). Hemolysis is shown as increase in absorbance at 405 nm. **B,** Cultured monocytic U937 cells were incubated with increasing concentrations of PLY with or without NanA (800 nM). Here, *C. perfringens* streptolysin O (SLO) that cannot lyse cells without a reducing agent was used as a negative control. Permeability is shown as percentage of cells stained positive for DAPI in flow cytometry. Error bars indicate SD calculated from at least two biological replicates. Binding of PLY to **(C)** rabbit HDL, **(C and D)** human total HDL, **(D)** HDL-2, HDL-3 and BSA coated microtiter plates was analyzed by ELISA as described in materials and methods. **(C)** Binding % is calculated as relative to 100% binding to HDL-2 at 33 nM PLY concentration. **(D)** The detected statistical significant differences ($p<0.05$) at 33 nM PLY concentration was calculated using one-way ANOVA followed by Dunnett's non-parametric multiple comparison test. Error bars indicate SD values calculated from three repeated experiments.

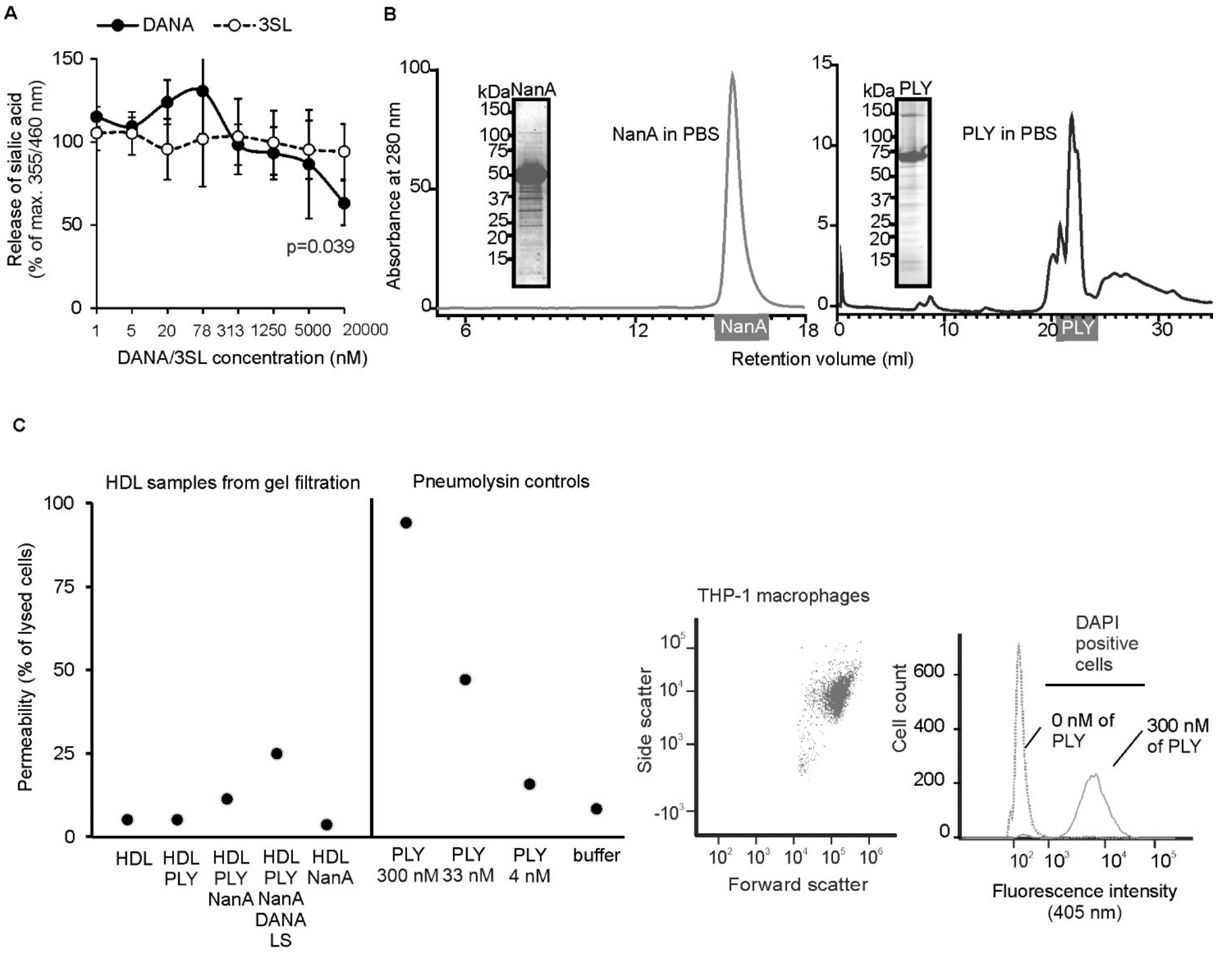

Figure S2. Activity of NanA inhibited by DANA and lytic activity of PLY in the HDL-2 fractions after gel filtration (Related to Figure 3). A, Inhibition of NanA using increasing concentrations of neuraminidase inhibitor 2,3-didehydro-2-deoxy-N-acetylneuraminic acid (DANA). Sialic acid analog 3'-Sialyllactose (3SL) showed no inhibitory activity towards NanA. Release of sialic acids is calculated as % of maximum absorbance measured at the lowest DANA/3SL concentration. The detected statistical significance ($p<0.05$) between highest and lowest NanA concentrations were calculated using Mann-Whitney U-test. Error bars showing SDs are calculated from at least three biological replicates. **B,** PLY and NanA were run in the same conditions as the HDL-2 samples in the study. NanA eluted at retention volumes between 14.5-15 ml, while PLY eluted at retention volumes between 20-23 ml. PLY and NanA run in SDS-PAGE with the kDa are shown with molecular weight marker. **C,** Cultured monocytic THP-1 cells were incubated with different concentrations of PLY and with 5 µg of the HDL-2 samples incubated with or without PLY, NanA, DANA and/or Liposomes (LS). Permeability is shown as percentage of lysed cells stained positive for DAPI in flow cytometry. Some cytolytic activity can be seen in the sample with PLY and NanA inhibitors when compared to the buffer background. (upper right) Density plot of THP-1 cells and (lower right) histogram of background cells (0 nM of PLY) and DAPI positive cells (300 nM of PLY) used for calculation of permeability % in FlowJo.

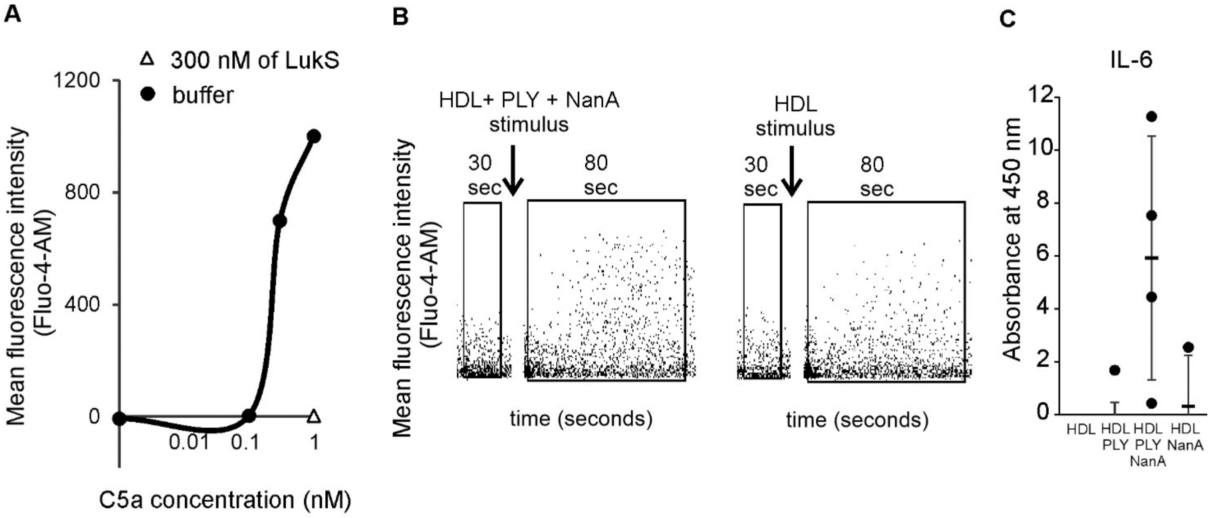

Figure S3. Calcium mobilization of HEK-C5aR cells (Related to Figure 4). A, Calcium mobilization induced by active C5a at 0-1 nM concentrations in the presence or absence of 300 nM LukS. **B,** Gating of Fluo-4-AM labeled HEK-C5aR cells before and after addition of the stimulus (HDL-2 particles incubated with or without PLY and NanA). **C,** Supernatants of HUVEC cell cultures incubated with HDL, HDL+PLY, HDL+PLY+NanA and HDL+NanA subjected to ELISA assay. IL-6 was released in the presence of HDL+PLY+NanA. The assay was repeated four times.

Table S1. Protein identification of plasma isolated total HDL separated from blood incubated with or without pneumolysin or pneumolysin and neuraminidase A (Related to Figure 1E).

Incubated with pneumolysin and neuraminidase A						
Accession	Description	*Score	Coverage	# Unique Peptides	# PSMs	MW [kDa]
**P02647**	Apolipoprotein A-I	261.39	76.40	30	138	30.8
**P02671**	Fibrinogen alpha chain	81.43	25.98	18	47	94.9
**P69905**	Hemoglobin subunit alpha	81.90	90.85	11	34	15.2
**P68871**	Hemoglobin subunit beta	75.17	81.63	8	32	16.0
**P02649**	Apolipoprotein E	28.20	36.91	13	22	36.1
**P02652**	Apolipoprotein A-II	46.58	72.00	9	21	11.2
**P02654**	Apolipoprotein C-I	19.62	42.17	7	20	9.3
**P0DJI8**	Serum amyloid A-1	46.87	56.56	5	20	13.5
**P01024**	Complement C3b	35.74	4.87	7	17	187.0
**P02042**	Hemoglobin subunit delta	31.35	69.39	4	16	16.0
**O14791**	Apolipoprotein L1	15.31	13.07	5	14	43.9
**P35542**	Serum amyloid A-4 protein	21.87	40.00	5	11	14.7
**P02655**	Apolipoprotein C-II	14.78	52.48	6	10	11.3
**P06727**	Apolipoprotein A-IV	9.69	25.76	10	10	45.4
**P02656**	Apolipoprotein C-III	30.95	37.37	4	9	10.8
**P10909**	Clusterin	12.27	16.26	8	9	52.5
**P01009**	Alpha-1-antitrypsin	1.77	14.59	6	9	46.7
**P0C0L4**	Complement C4-A	12.39	4.64	5	9	192.7
**P27169**	Serum paraoxonase/arylesterase 1	11.68	24.23	8	9	39.7
**P02675**	Fibrinogen beta chain	20.45	4.07	2	8	55.9
**P02766**	Transthyretin	11.27	49.66	6	6	15.9
**O95445**	Apolipoprotein M	5.88	22.87	5	5	21.2
**Q14624**	Inter-alpha-trypsin inhibitor heavy chain H4	2.96	5.16	3	4	103.3
**P02775**	Platelet basic protein	12.28	11.72	2	4	13.9
**P02768**	Albumin	0.00	5.58	3	4	69.3
**Pneumolysin****	sequence	8.47	13.40	4	4	52.7
**P04114**	Apolipoprotein B-100	2.04	0.68	2	2	515.3
**P04180**	Phosphatidylcholine-sterol acyltransferase	3.50	9.09	2	2	49.5
**P00739**	Haptoglobin-related protein	3.07	5.17	1	1	39.0
**Q9BUN1**	Protein MENT	2.99	4.40	1	1	36.7
**Incubated with buffer**						
Accession	Description	*Score	Coverage	# Unique Peptides	# PSMs	MW [kDa]
**P02647**	Apolipoprotein A-I	308,22	78,65	37	164	30,76
**P02671**	Fibrinogen alpha chain	56,47	18,24	14	33	94,91
**P02652**	Apolipoprotein A-II	55,14	72	9	26	11,17
**P02649**	Apolipoprotein E	39,56	34,38	14	24	36,13
**P02654**	Apolipoprotein C-I	18,13	42,17	7	18	9,33
**P35542**	Serum amyloid A-4 protein	23,56	53,85	8	16	14,74
**P02656**	Apolipoprotein C-III	36,89	58,59	7	14	10,85
**P06727**	Apolipoprotein A-IV	11,10	32,58	13	13	45,37
**P02655**	Apolipoprotein C-II	17,18	56,44	7	12	11,28
**P0C0L5**	Complement C4-B	15,57	8,94	1	12	192,63

**P0C0L4**	Complement C4-A	13,55	9,06	1	12	192,66
**P0DJI8**	Serum amyloid A-1 protein	44,57	56,56	3	12	13,52
**P27169**	Serum paraoxonase 1	11,48	28,17	8	11	39,71
**O14791**	Apolipoprotein L1	13,82	15,08	6	9	43,95
**P69905**	Hemoglobin subunit alpha	18,55	30,28	5	9	15,25
**P04114**	Apolipoprotein B-100	11,16	1,97	8	8	515,28
**P10909**	Clusterin	9,49	16,26	7	7	52,46
**P0DJI9**	Serum amyloid A-2 protein	16,75	36,07	1	7	13,52
**P68871**	Hemoglobin subunit beta	9,81	36,05	5	7	15,99
**P02675**	Fibrinogen beta chain	13,28	4,07	2	6	55,89
**P01024**	Complement C3	6,06	3,25	4	6	187,03
**P00739**	Haptoglobin-related protein	2,22	19,25	5	6	39
**P02768**	Serum albumin	1,72	6,90	4	6	69,32
**P55056**	Apolipoprotein C-IV	6,97	28,35	4	4	14,54
**P01009**	Alpha-1-antitrypsin	6,28	9,33	3	4	46,71
**P02766**	Transthyretin	4,27	24,49	3	3	15,88
**O95445**	Apolipoprotein M	2,82	11,17	3	3	21,24
**Q13790**	Apolipoprotein F	5,45	4,29	1	2	35,38
**Q9BUN1**	Protein MENT	3,50	6,74	2	2	36,75
**P80108**	Phosphatidylinositol-glycan-specific phospholipase D	1,84	3,33	2	2	92,28
**P02775**	Platelet basic protein	3,12	17,19	2	2	13,89
**A8MWD9**	Putative small nuclear ribonucleoprotein G-like protein 15	0	26,32	1	1	8,54
**P0CG39**	POTE ankyrin domain family member J	0	0,96	1	1	117,31
**Q15465**	Sonic hedgehog protein	0	3,46	1	1	49,58
**Q8WZA9**	Immunity-related GTPase family Q protein	0	3,37	1	1	62,68
**P13747**	HLA class I histocompatibility antigen, alpha chain E	1,95	3,91	1	1	40,03
**Q8TF61**	F-box only protein 41	1,80	1,03	1	1	94,44
**P04439**	HLA class I histocompatibility antigen, A alpha chain	1,98	3,84	1	1	40,82
**O95810**	Caveolae-associated protein 2	1,97	3,29	1	1	47,14
**P01034**	Cystatin-C	1,95	7,53	1	1	15,79
**Incubated with pneumolysin**						
Accession	Description	*Score	Coverage	Unique Peptides	# PSMs	MW [kDa]
**P02647**	Apolipoprotein A-I	254,62	78,65	31	136	30,76
**P02671**	Fibrinogen alpha chain	82,83	20,44	19	50	94,91
**P68871**	Hemoglobin subunit beta	56,68	81,63	6	23	15,99
**P69905**	Hemoglobin subunit alpha	47,70	70,42	9	21	15,25
**P02649**	Apolipoprotein E	31,45	33,44	13	20	36,13
**P02654**	Apolipoprotein C-I	21,87	42,17	7	16	9,33
**P0DJI8**	Serum amyloid A-1	39,72	59,02	4	16	13,52

**P06727**	Apolipoprotein A-IV	18,46	32,58	13	15	45,37
**P02652**	Apolipoprotein A-II	27,01	69	7	15	11,17
**P0C0L4**	Complement C4-A	19,32	5,96	7	13	192,66
**P02656**	Apolipoprotein C-III	23,53	39,39	5	9	10,85
**P01024**	Complement C3	17,31	4,81	5	9	187,03
**P35542**	Serum amyloid A-4 protein	16,57	40	5	9	14,74
**P02042**	Hemoglobin subunit delta	13,64	42,18	1	9	16,05
**O14791**	Apolipoprotein L1	12,64	10,80	4	8	43,95
**P0DJI9**	Serum amyloid A-2	16,54	36,07	1	8	13,52
**P02675**	Fibrinogen beta chain	19	4,07	2	7	55,89
**P10909**	Clusterin	7,87	9,80	6	7	52,46
**P27169**	Serum paraoxonase 1	9,39	24,23	7	7	39,71
**P02766**	Transthyretin	7,52	41,50	5	6	15,88
**P02655**	Apolipoprotein C-II	1,79	28,71	3	5	11,28
**O95445**	Apolipoprotein M	4,72	22,34	3	4	21,24
**P02100**	Hemoglobin subunit epsilon	5,63	21,77	1	4	16,19
**P02768**	Serum albumin	1,77	3,94	2	4	69,32
**P02775**	Platelet basic protein	8,93	11,72	2	3	13,89
**Pneumolysin****	sequence	9,28	10,21	3	3	52,74
**Q13790**	Apolipoprotein F	1,85	4,29	1	2	35,38
**P01009**	Alpha-1-antitrypsin	3,62	4,78	2	2	46,71
**Q15465**	Sonic hedgehog protein	2,04	5,41	2	2	49,58
**P49913**	Cathelicidin antimicrobial peptide	1,74	5,88	1	1	19,29
**P55056**	Apolipoprotein C-IV	0	8,66	1	1	14,54
**O60503**	Adenylate cyclase type 9	2,91	2	1	1	150,60
**P00739**	Haptoglobin-related protein	1,70	3,74	1	1	39
**Q15149**	Plectin	0	0,19	1	1	531,47
**Q9BUN1**	Protein MENT	2,93	4,40	1	1	36,75
**P80108**	Phosphatidylinositol-glycan-specific phospholipase D	2,45	1,55	1	1	92,28
**P61769**	Beta-2-microglobulin	2,11	10,92	1	1	13,71
**P01034**	Cystatin-C	1,69	7,53	1	1	15,79

* Indicates relevance of the protein. Calculated by Proteome Discoverer software according to the score thresholds
# Numbers shown as peptide spectrum matches (PSMs) presenting the total number of identified peptide spectra matched for the protein.
**Recombinant *S. pneumoniae* pneumolysin
